# Ambient fine particulate matter inhibits 15-lipoxygenases to promote lung carcinogenesis

**DOI:** 10.1186/s13046-019-1380-z

**Published:** 2019-08-16

**Authors:** Ming-Yue Li, Li-Zhong Liu, Wende Li, Calvin S. H. Ng, Yi Liu, Angel W. Y. Kong, Zhili Zhao, Shanshan Wang, Haolong Qi, Hao Jia, Shucai Yang, Jing Du, Xiang Long, Rocky L. K. Ho, Ernest C. W. Chak, Innes Y. P. Wan, Tony S. K. Mok, Malcolm J. Underwood, Nirmal Kumar Gali, Zhi Ning, George G. Chen

**Affiliations:** 1 Department of Surgery, Head and Neck Surgery, The Chinese University of Hong Kong, Prince of Wales Hospital, Shatin, N.T Hong Kong; 2 Faculty of Medicine, Shenzhen University Health Science Center, Shenzhen University, Shenzhen, China; 3 Shenzhen Research Institute, The Chinese University of Hong Kong, Shenzhen, Guangdong China; 4 Division of Environment and Sustainability, The Hong Kong University of Science and Technology, Clear Water Bay, Kowloon, Hong Kong; 5 Department of Clinical Oncology, Head and Neck Surgery, The Chinese University of Hong Kong, Prince of Wales Hospital, Shatin, N.T Hong Kong; 6 Guangdong Key Laboratory of Laboratory Animal, Guangdong Laboratory Animals Monitoring Institute, Guangzhou, China; 7 Guangdong Medical College, Zhangjiang, Guangdong China; 8 Department of Clinical Laboratory, Pingshan District People’s Hospital of Shenzhen, Shenzhen, China; 9 Peking University Shenzhen Hospital, Shenzhen, Guangdong China; 10 Department of Otorhinolaryngology, Head and Neck Surgery, The Chinese University of Hong Kong, Prince of Wales Hospital, Shatin, N.T Hong Kong

**Keywords:** Lung cancer, PM_2.5_, NNK, 15-lipoxygenases (15-LOXs), Epigenetic and post-translational regulation

## Abstract

**Background:**

Epidemiological observations have demonstrated that ambient fine particulate matter with *d*_p_ < 2.5 μm (PM_2.5_) as the major factor responsible for the increasing incidence of lung cancer in never-smokers. However, there are very limited experimental data to support the association of PM_2.5_ with lung carcinogenesis and to compare PM_2.5_ with smoking carcinogens.

**Methods:**

To study whether PM_2.5_ can contribute to lung tumorigenesis in a way similar to smoking carcinogen 4-methylnitrosamino-l-3-pyridyl-butanone (NNK) via 15-lipoxygenases (15-LOXs) reduction, normal lung epithelial cells and cancer cells were treated with NNK or PM_2.5_ and then epigenetically and post-translationally examined the cellular and molecular profiles of the cells. The data were verified in lung cancer samples and a mouse lung tumor model.

**Results:**

We found that similar to smoking carcinogen NNK, PM2.5 significantly enhanced cell proliferation, migration and invasion, but reduced the levels of 15-lipoxygenases-1 (15-LOX1) and 15-lipoxygenases-2 (15-LOX2), both of which were also obviously decreased in lung cancer tissues. 15-LOX1/15-LOX2 overexpression inhibited the oncogenic cell functions induced by PM2.5/NNK. The tumor formation and growth were significantly higher/faster in mice implanted with PM2.5- or NNK-treated NCI-H23 cells, accompanied with a reduction of 15-LOX1/15-LOX2. Moreover, 15-LOX1 expression was epigenetically regulated at methylation level by PM2.5/NNK, while both 15-LOX1 and 15-LOX2 could be significantly inhibited by a set of PM2.5/NNK-mediated microRNAs.

**Conclusion:**

Collectively, PM2.5 can function as the smoking carcinogen NNK to induce lung tumorigenesis by inhibiting 15-LOX1/15-LOX2.

**Electronic supplementary material:**

The online version of this article (10.1186/s13046-019-1380-z) contains supplementary material, which is available to authorized users.

## Background

Although cigarette smoking is still a major causing factor for lung cancer, the incidence of lung cancer without history of tobacco smoking is increasing [1, 2]. Global statistics indicates that lung cancer in never smokers (LCNS) accounts for about 25% of lung cancer worldwide [3]. Among the factors that contribute to the development of LCNS, the polluted air, especially PM_2.5_ (*d*_p_ < 2.5 μm), has been indicated as the major factor [3–6]. PM_2.5_ exposures after lung cancer diagnosis also shorten patients’ survival [7]. The International Agency for Research on Cancer has concluded that exposure to PM_2.5_ causes lung cancer [8].

The acquisition of cellular characteristics of lung cancer malignancy is critically linked to abnormal cell proliferation, formation of cancer stem cells (CSC), and epithelial mesenchymal transition (EMT) alterations, resulting in tumor growth, invasion and metastasis [9, 10]. Despite the epidemiological observations that strongly implicate air pollution in the development of LCNS, there have been very limited experimental studies to verify the epidemiological finding. A recent report showed that PM_2.5_ upregulated autophagy, migration, invasion and EMT to promote lung cancer progression via inducing IncRNA LCPAT1 and regulator of chromosome condensation 2 (RCC2) [11]. PM_2.5_ is also demonstrated to cause epigenetic and microenvironmental alterations in lung cancer, including tumor-associated signaling pathway activation mediated by microRNA dysregulation, DNA methylation, and increased levels of cytokines and inflammatory cells [6].

15-Lipoxygenases (15-LOXs), 15-LOX1 and 15-LOX2, appear to be tissue-specific expression and are found in human lung cells [12]. The metabolites of 15-LOXs can function as endogenous ligands of peroxisome proliferator-activated receptor-γ (PPARγ) [12]. Our studies have amply demonstrated that the activation of PPARγ can significantly inhibit the growth of lung cancer and the downregulation of PPARγ activity contributes to lung cancer progression [13]. Furthermore, recovering the loss of 15-LOXs could effectively prevent lung tumorigenesis caused by the major smoking carcinogen 4-methylnitrosamino-l-3-pyridyl-butanone (NNK), induce apoptosis and arrest tumor cell growth through activation of PPARγ [14, 15]. The expression of 15-LOXs in lung cancer cells is very low or even undetectable [12, 13, 16]. The reduction of 15-LOXs occurs much earlier than the appearance of NNK-induced mouse lung tumors, strongly indicating a positive role of 15-LOX downregulation in the smoking-induced lung carcinogenesis in mice [12, 13]. All these findings support the idea that 15-LOXs are tumor suppressors in smoking carcinogen NNK-induced lung tumorigenesis. The loss of 15-LOXs may contribute to the development of lung cancer, whereas their presence may inhibit it [12, 15, 16]. The reduction of 15-LOXs can lead to the hypoproduction of their metabolites, which inactivates PPARγ and facilitates lung carcinogenesis. However, there is no study on whether 15-LOXs participate in the PM_2.5_-induced lung carcinogenesis. This study shed new light on how PM_2.5_ induced lung carcinogenesis via inhibiting 15-LOXs.

## Materials and methods

### Reagents

Fetal bovine serum (FBS), Cell Dissociation Reagent, Dulbecco’s modified Eagle medium (DMEM), LHC-9, and RPMI medium 1640 were provided by Invitrogen (Carlsbad, CA). Cancer Stem Cell Media Premium™ was purchased from ProMab Biotechnologies (Richmond, CA). The antibody against VECTOR ImmPRESS Anti-Goat Ig was provided by Santa Cruz Biotechnology (Santa Cruz, CA). Antibodies against CD133 were provided by Proteintech (#18470–1-AP, Rosemont, IL). Antibodies against 15-LOX2 were provided by lifespan (#LS-B1588). Antibodies against ALDH1A1 (#ab23375), 15-LOX1 (#ab80221), Protein Block solution and DAB substrate were provided by Abcam (Cambridge, MA). Antibodies against Bcl-2 (##4223), Bax (#14796S), pGSK3β (#5558 s), GSK3β (#12456 s), Nanog (#4903), TCF4 (#2569 s) and β-catenin (#8480 s) were purchased from Cell Signaling (Boston, MA). ECL reagent kit was purchased from GE Healthcare Life Sciences (Piscataway, NJ). 3-(4,5-Dimethylthiazol-2-yl)-2,5-Diphenyltetrazolium Bromide (MTT) was purchased from Sigma (St, Louis, MO). Ultra-low-adhesion 6 well plates and 10 mm plates were purchased from Corning (Tewksbury, MA).

### PM_2.5_ sample collection and water extraction

The PM_2.5_ was collected at Kowloon Tong of Hong Kong [17]. In brief, Personal Cascade Impactor Samplers (PCIS, SKC Inc., Eighty-Four, PA) were used to collect PM_2.5_ on 37 mm Teflon filter (PALL Life Sciences, Ann Arbor, MI). Samples were collected lasting for 7 days. Each PCIS was run at flow rate of 9 l per minute which was checked regularly with a Gillian air flow calibrator (Sensidyne Inc., Clearwater, FL) during the week long sampling period. Field blanks for Teflon filters were also collected during sampling period. All filters were conditioned for 48 h in a temperature- and relative humidity-controlled environment. The PM_2.5_ mass concentrations were determined by weighing the filters with a microbalance prior to PM extraction from filters. For water extraction, the filters were cut into pieces and soaked in 8 mL of Milli-Q water in 15 mL metal free centrifuge tubes, then extracted by vortex–assisted shaking using a multi-tube vortex mixer (Model X-2500, VWR). After 12 h of vortex, the extracts were filtered with 0.22 μm filter membranes and stored at − 20 °C.

### Cell culture and cell proliferation assay by MTT

Lung cancer cell line NCI-H23 was purchased from the American Type Culture Collection (ATCC). Bet1A is lung normal bronchial epithelial cells (gift of J. E. Lechner, Laboratory of Human Carcinogenesis, National Cancer Institute) [18–21]. Though Bet1A cells had extended culture lifespans compared to normal human bronchial epithelial cells, the cells retained features of epithelial cells [18–21]. The NCI-H23 cells were cultured in DMEM supplemented with 10% inactivated FBS and incubated at 37 °C under 5% CO_2_. The Bet1A cells were cultured in medium LHC-9. In sphere culture system, cells were cultured in Cancer Stem Cell Media Premium to generate spheroid forming cancer stem cells (CSCs). The proliferation of cells treated under various concentrations of PM_2.5_ was determined by MTT assay [15]. Various concentrations of PM_2.5_ (5 μg/ml, 10 μg/ml, 25 μg/ml, 50 μg/ml, 75 μg/ml), or 10 μM NNK [22] were added to the cells. The medium with filter blank extraction was added as a control. The cells were incubated for 24, 120, and 168 h. The concentrations of the PM_2.5_ increasing cell growth significantly were determined from cell survival plots. For long-term period treatment, 5 μg/ml PM_2.5_ or 10 μM NNK were used to treat the cells for 28 days. Then the cells were seeded in 96-well plates and cultured for 24 and 48 h. Based on MTT results, Cells were studied in the presence of 5 μg/ml PM_2.5_ or 10 μM NNK for a short-term period of several hours to several days, and for long-term period treatment of 28 days. Non-treatment cells were set up as the control. Lung cancer cells A549, NCI-H460 and NCI-H1975 purchased from ATCC were also cultured to confirm the PM2.5 effect.

### Human lung tissues

In total, 109-Paired human primary non-small cell lung cancers (NSCLCs) and adjacent normal lung tissues were collected immediately after surgical resection at the Prince of Wales Hospital (Hong Kong, China). Human ethics approval was obtained from the joint Chinese University of Hong Kong-New Territories East Cluster Clinical Research Governance and Management Committee. Of the 109 patients, 34 were current cigarette smokers with an average smoking history of 35 years, 36 patients were previous cigarette smokers with an average smoking history of 28 years, and the other 39 patients were non-smokers. All tumor and non-tumor tissue specimens were confirmed by histological examination. The specimens were snap-frozen in liquid nitrogen and stored at 80 °C and were also fixed in 10% formalin and embedded in histochemical staining examination.

### Sphere formation assay

NCI-H23 and Bet1A cells were treated with 5 μg/ml PM_2.5_ or 10 μM NNK for 6 h. Cells were dissociated into single-cell suspension and plated in ultra-low adhesion 6 well plates at the density of 2500 cells/ml in Cancer Stem Cell Media Premium and allowed to grow for 10 days. Images of the first-generation tumor spheres formed were taken using phase contrast microscope (Nikon) and total number and size of spheres more than 60 *μ* m were counted. The first-generation tumor sphere cells were dissociated into single-cell suspension by Cell Dissociation Reagent. Cells were cultured to obtain second-generation spheres. Tumor spheres were counted to assess the self-renewal of CSCs.

### Wound healing assay

To assess cell motility, NCI-H23 cells or Bet1A cells treated by PM_2.5_ or NNK for 28 days (5 × 10^5^ cells/ mL) were seeded in 24-well plates (Corning, New York) and cultured as confluent monolayers. The cells were incubated with 10 μg/ml mitomycin-C (Sigma, MO) for 2 h and then starved in serum-free medium for 24 h to suppress proliferation. Non-treated cells were set up as the control. The monolayers of NCI-H23 were scraped with a sterile 1000-μl micropipette tip (0 h) and Bet1A were scraped with a sterile 200-μl micropipette tip (0 h) to create a denuded zone with a constant width and were washed twice with phosphate-buffered saline (PBS) to remove cellular debris. The scratched monolayers were imaged for 24 h, 48 h, and 72 h using an inverted microscope (Olympus, Japan) at 200× magnification in a blinded fashion. The relative percentage of wound healed was analysis by Image J software.

### Invasion assay

Cell invasion was determined using BD BioCoat Matrigel Invasion Chamber (BD Biosciences) according to the instruction of the manufacturer. Briefly, NCI-H23 cells or Bet1A cells treated by PM_2.5_ or NNK for 28 days were incubated with 10 μg/ml mitomycin-C (Sigma, MO) for 2 h and then starved in serum-free medium for 24 h to suppress proliferation. The cells (2 × 10^4^ cells/well) were seeded onto the top chamber in serum-free cell culture medium. Complete culture medium (supplemented with 10% FBS) was added to the bottom chamber as a chemoattractant. After 48 h, cells that had invaded through the membrane were stained with 0.1% Crystal violet. Migrated cells in randomly selected fields were observed by light microscopy (Olympus, Japan) at a magnification of 400 × .

### Plasmid DNA and transfection

The plasmids for wild-type human 15-LOX-1 and 15-LOX-2 were generous gifts from Professor Alan R. Brash (Vanderbilt University School of Medicine). The X-tremeGENE® HP Transfection reagent (#636546001, Roche, Basel, Switzerland) was used to transfect plasmids into cells according to the manufacturer’s instructions. Cells transfected with the empty vector were used as the control.

### Immunohistochemistry

Immunohistochemical staining of 15-LOX1, 15-LOX2 and vimentin were performed for 109 pairs of human lung tissues as described previously [13].

### Fluorescence-immunohistochemical staining and microscopy

Fluorescence-immunohistochemical staining for vimentin was performed and the stained cells were examined using the Zeiss Spot imaging system (Carl Zeiss, Jena, Germany) [23]. For detecting the cell surface Vimentin, the cells grown on glass coverslips were fixed and not permeabilized before incubation with primary antibody. The stained cells were examined using the Zeiss Spot imaging system (Carl Zeiss, Jena, Germany).

### MassArray for methylation assay

MassArray for methylation assay (BGI, China) of genes was employed to detect the 15-LOX1 and 15-LOX2 gene promotor methylation levels. The software, www.ebi.ac.uk/Tools/seqstats/emboss_cpgplot/, was used to predict the CpG islands from the upstream of 5000 bp of start codon to downstream of 1000 bp start codon of genes (Additional file 1: Figure S3A). One CpG island in 15-LOX1 promotor region was found (Additional file 1: Figure S3B). No CpG island was predicted in 15-LOX2 gene. Sequenom®EpiDesigner process was used to design plans for 15-LOX1 gene methylation assay (Additional file 1: Figure S3C).

### Real-time PCR

For 15-LOX expression assay, total RNA was extracted for real-time PCR using SYBR Green qPCR SuperMix (Invitrogen) according to our previous work [12]. Briefly, total RNA was extracted using Trizol reagent (Invitrogen, Grand Island, NY) according to the manufacturer’s protocol. cDNA was synthesized from 2 μg total RNA using a high capacity cDNA reverse transcription kit (Promega, Madison, WI). Aliquots of cDNA were used as template for real-time PCR reactions containing gene-specific primers and SYBR Green qPCR SuperMix. Real-time PCR was performed using the ABI Prism 7900 detection system (Applied Biosystems, Carlsbad, CA). The expression of target genes in the treatment and control groups was normalized using the house-keeping gene GAPDH and the fold change in the expression of each target gene was calculated. The following primer sequences for the targeted regions of the 15-LOX-1 (295 bp), 15-LOX-2 (170 bp), and glyceraldehyde-3-phosphate dehydrogenase (GAPDH) (136 bp for human) genes were used as our previous study [12]: 15-LOX-1, (forward) 5′-GCCCAGGACCGAGGGTTTC − 3′ and (reverse) 5′-GGCCCACAGCCACCATAAC-3′; 15-LOX-2, (forward) 5′-TTGCAGGCTGAGCTAGAGAAGG-3′ and (reverse) 5′-CTG AGCTGGATGGCGAGAGG-3′; 12/15-LOX (forward) 5′-ATCGGTACGTGGGAATG-3′, 12/15-LOX (reverse) 5′-GCGCAGTGCATGTGAAGATG-3′; human GAPDH (forward) 5′-GGGTGTGAACCATGAGAA-3′ and (reverse) 5′-GACTGTGGTCATGAGTCCT-3′. Real-time PCR reactions consisted of the following steps: 50 °C 2 min, 95 °C 10 min, 38 cycles at 95 °C 15 s and 60 °C 60 s.

The expression of miR-18a, miR-125b, miR-203, miR-20a, miR-20b, miR-17, miR-106a, miR-106b, miR-93, miR-590-3p (# E01007) and U6 (#E01008) was detected using Hairpin-it™ miRNAs qPCR Quantitation Kit (Genepharma, China). cDNA was synthesized from 1.5 μg total RNA under RT Reaction Program at 30 min at 25 °C, 30 min at 42 °C followed by 5 min at 85 °C. Real-time PCR reactions consisted of the following steps: 95 °C for 3 min, then 40 cycles at 12 s at 95 °C and 40 s at 62 °C. The expression of target miRNAs in the treatment and control groups was normalized using U6 and the fold change in the expression of each target gene was calculated.

### Reporter assays of 3′-UTR-luciferase plasmid of 15-LOX1 and 15-LOX2

The Luc-15LOX1/2wt with full-length 3′-untranslated region (UTR) of 15-LOX1 (674 nt) and 15-LOX2 (545 nt) were cloned into pGL3-promoter-vector (Additional file 1: Figure S4). For the reporter assay, NCI-H23 and Bet1A cells transiently transfected with reporter plasmids using the X-tremeGENE® HP Transfection reagent (#636546001, Roche, Basel, Switzerland). The pGL3 basic vector and the pGL3 control were used as negative and positive controls, respectively. Twenty-four hours later, the cells were treated with PM_2.5_ or NNK for a specific period. Non-treated cells were set up as control. Reporter assays were performed using the Dual-luciferase assay system (Promega, Madison, WI), normalized for transfection efficiency by co-transfected Renilla luciferase.

### Xenograft model

A subcutaneous (S.C.) tumor model was set up in nude mice to determine the tumorigenesis of lung cells treated with PM_2.5_ or NNK for 28 days. All experimental procedures were approved by the Animal Ethics Committee of the Chinese University of Hong Kong (Hong Kong, China). Briefly, cells (5 × 10^6^) were S.C. implanted into the left and right dorsal flank of 5-week-old female BALB/C athymic (nu/nu, n = 9/group) nude mice, respectively. After cell implantation, the general health, body weight, and activity of the mice were monitored daily. Tumors were measured weekly, in two dimensions by external caliper and Tumor volume (V) was estimated by measuring the longest diameter (L) and shortest diameter (W) of the tumor and calculated by formula [length x width (mm)^2^]/2 [24]. The size of tumor was monitored for 6 weeks. At the endpoint, tumors were harvested and measured.

### Western-blot

The protein isolated was performed western blot as described previously [22].

### Statistical analysis

Continuous data were expressed as mean ± SEM (continuous variables) or described as frequency and percentage (categorical data). The difference was determined by ANOVA with repeated-measures ANOVA. To compare the difference between two groups, independent sample t test or Mann–Whitney U test was used. Based on the 15-LOX1/15-LOX2/Vimentin expression levels in tumor tissues and the paired non-tumor tissues, the expression level was graded. When the expression of 15-LOX1/15-LOX2.Vimentin level in each paired samples was considered, the expression in non-tumor tissue was set up as the normal, and the expression in tumor tissue was set as low/high expression when the level of the expression was less/more than that of in the non-tumor tissue. The clinic-pathologic features in patients with relative expressing 15-LOX1/15-LOX2/Vimentin were compared using Pearson’s chi-squared test or Fisher’s exact test for categorical variables. All the statistical analyses were performed using GraphPad Prism, version 6.0 (GraphPad Software) or SPSS, version 20.0 (SPSS Inc.). P < 0.05 was considered statistically significant.

## Results

### PM_2.5_ or NNK regulates lung cell proliferation, CSC formation, cell migration, and invasion

**Fig. 1:**
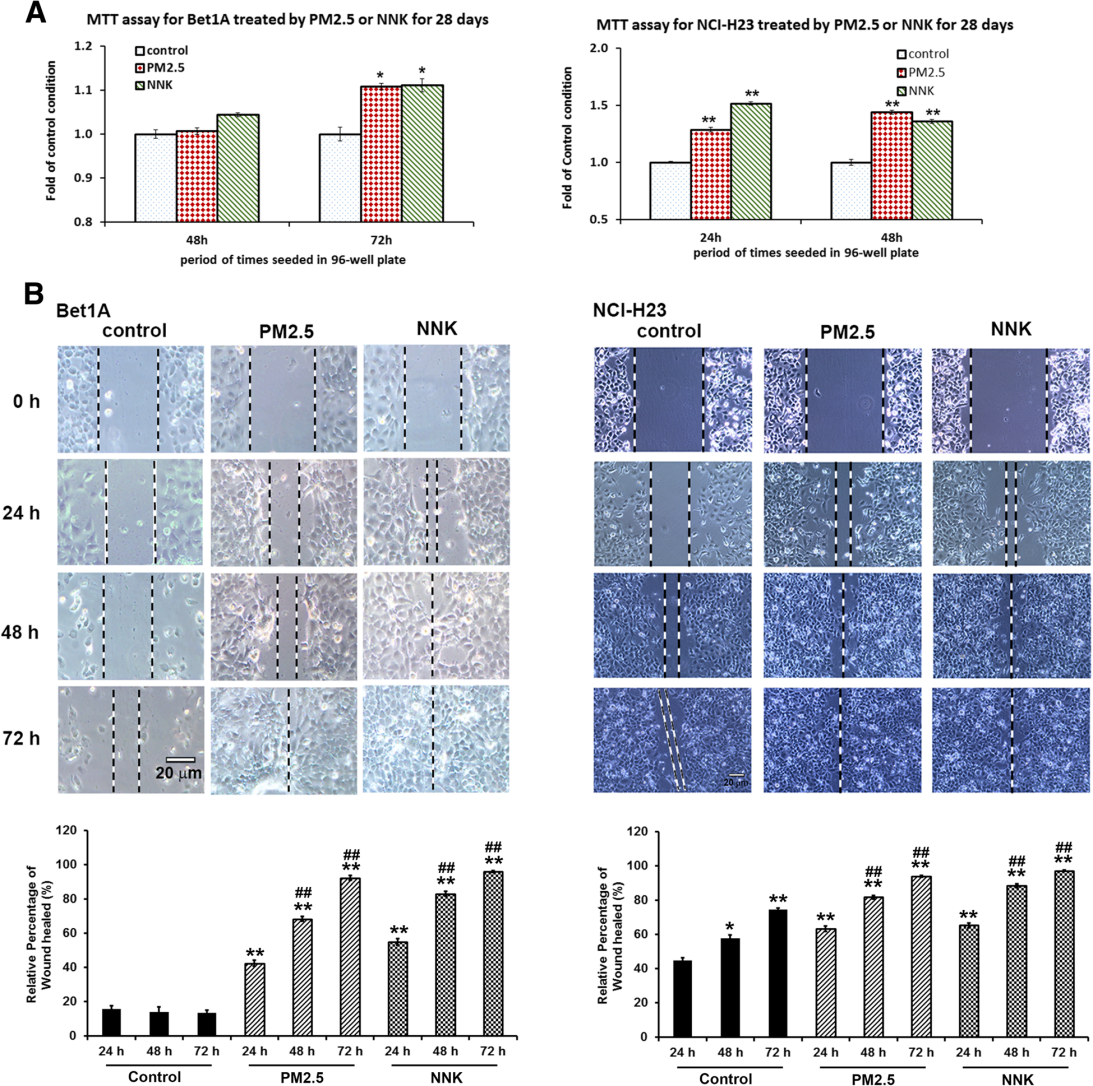
Induction of lung cell carcinogenesis by PM2.5 or NNK treatment. **a** PM2.5 or NNK treatment with 28 days promoted cell proliferation. Cells treated with 5 μg/ml PM2.5 or 10 μM NNK for 28 days were seeded for MTT assay. (*P < 0*.*05, ** *P < 0.01, n* = 4). **b** PM2.5 induced Bet1A and NCI-H23 cell migration. Cells treated by PM2.5 or NNK for 28 days were seeded for different periods. Images were taken using phase contrast microscope (Nikon) (scale bar, 20 μm). The relative percentage of wound healed was expressed as mean ± SD of three independent experiments. *p < 0.05 and **p < 0.01 vs control 24 h condition; ^##^*p* < 0.01 when PM_2.5_ or NNK 48 h and 72 h vs control 48 h and 72 h respectively

In light of the association between PM_2.5_ and lung carcinogenesis [3, 4, 8–10], we ask whether PM_2.5_ can function like NNK to affect cell proliferation, CSC formation, migration, and invasion. It was found that both PM_2.5_ and NNK could similarly exert positive effects on cell proliferation (Fig. 1a and Additional file 1: Figure S1A). The results showed that PM_2.5_ significantly increased the proliferation of the Bet1A cells but not NCI-H23 cells when the cells were treated with PM_2.5_ in a relatively short period within one week (Additional file 1: Figure S1A). It appeared that PM_2.5_ at 5 μg/ml or more or 10 μM NNK could significantly enhanced Bet1A cell proliferation. We found that 10 μM NNK could increase the proliferation of Bet1A cells (Additional file 1: Figure S1A) as well as NCI-H23 cells [22]. When the cells were treated by 5 μg/ml PM_2.5_ or 10 μM NNK for a longer period of 28 days, the cells exhibited significantly higher proliferation property than the non-treated control cells (Fig. 1a). Sphere formation assay for detection of CSC formation showed the increase in the number and size of stem cell spheres significantly in PM_2.5_- or NNK-treated lung cells (Additional file 1: Figure S1B and C). For the self-renewable ability of the CSCs, it showed that CSCs of NCI-H23 cells exhibited more vigorous self-development than that of Bet1A CSCs, since the tumor sphere formed by the second generation of NCI-H23 CSCs was significantly higher than its 1st generation’s CSCs (Additional file 1: Figure S1C). Wound healing assay and trans-well test results showed that PM_2.5_ and NNK increased the cell migration (Fig. 1b) and invasion (Additional file 1: Figure S1D).

### PM_2.5_ could function like NNK to induce/activate molecules related to cell proliferation, CSC formation, EMT and Wnt/β-catenin signal pathway

**Fig. 2:**
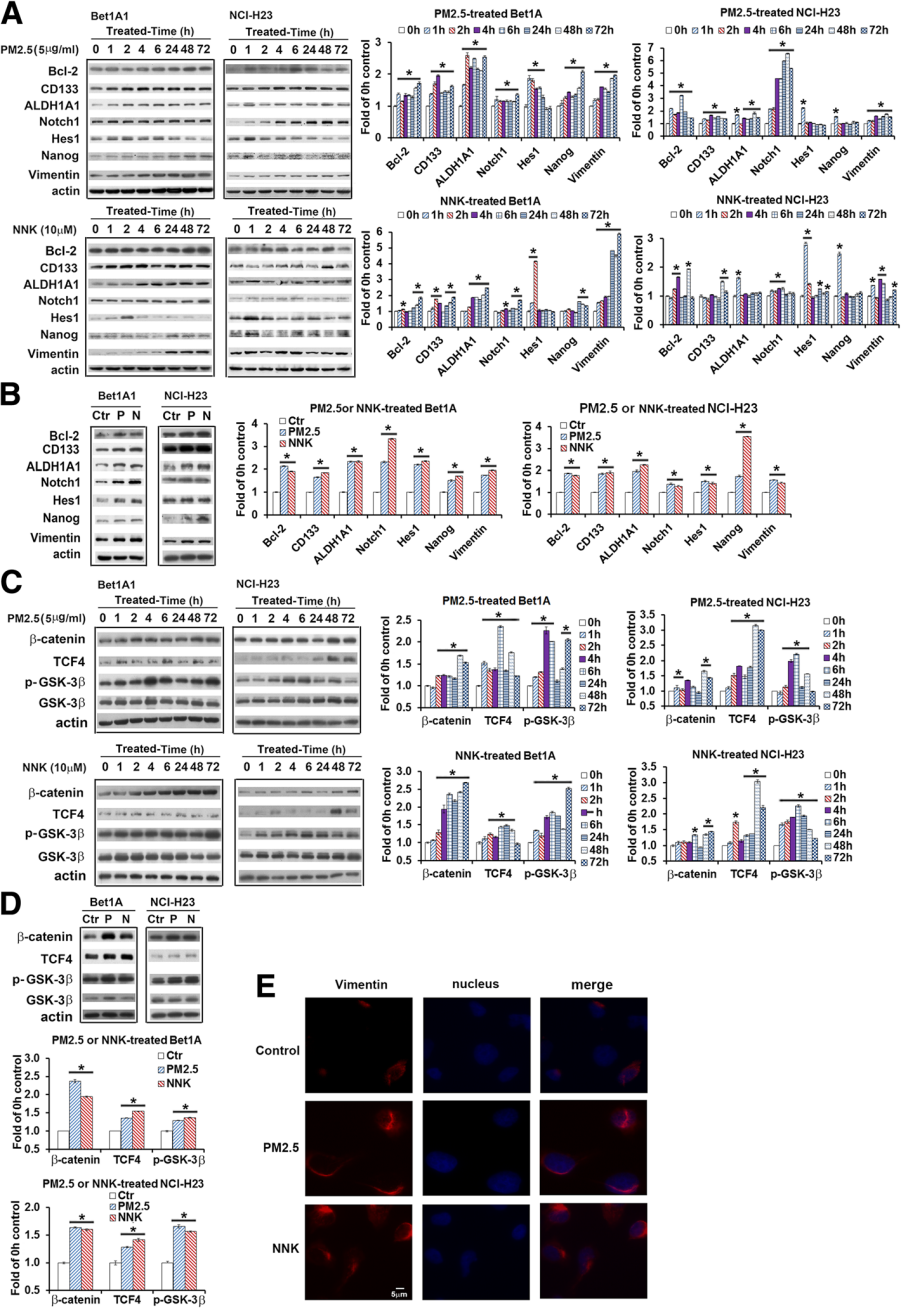
Effects of PM2.5 on oncogenic protein expression in Bet1A1 and NCI-H23 cells. **a** and (**b**) Effects of PM2.5 or NNK on lung carcinogenesis-related biomarkers; or (**c**) and (**d**) NNK or PM2.5 stimulated Wnt/GSK3β/β-catenin pathway. Cells were treated by 5 μg/ml PM2.5 or 10 μM NNK for 0 h, 1 h, 2 h, 4 h, 6 h, 24 h, 48 h and 72 h respectively (**a** and **c**). Cells of 0 h were the control condition. Or cells were treated with 5 μg/ml PM2.5 or 10 μM NNK for 28 days (**b** and **d**). Non-treated cells were cultured for 28 days as the control condition. Bcl-2, CD133, ALDH1A1, Notch1, Hes1, Nanog, vimentin, pGSK3β, GSK3β, β-catenin and TCF4 were detected by western blot. The quantification of protein was carried out by densitometry analysis and expressed as mean ± SE. The relative intensity of protein bands was summarized by column figure (*n* = 3, Ctr: non-treatment control; P: PM2.5; N: NNK; *p < 0.05 when compared each PM2.5- or NNK-treated condition with the control condition). **e** PM2.5 and NNK treatment increased vimentin expression. Bet1A Cells were treated with PM2.5 or NNK for 28 days. Vimentin antibody was used in connection with Rhodamine red-conjugated second antibody. DAPI (blue signal) was used for the counterstaining of the nucleus. The images were photographed (original magnification 400×, scale bar, 5 μm)

Both PM_2.5_ and NNK could upregulate the expression of Bcl-2, a proliferation-related protein [22]. The levels of lung CSCs markers, aldehyde dehydrogenase isoform 1A1 (ALDH1A1), CD133, Nanog, Notch1 and Hes1 [25], were markedly induced by either a shorter period of treatment (Fig. 2a) or a longer term of 28-day treatment (Fig. 2b). The expression of vimentin, a well-characterized biomarker of EMT marker [26], was also increased during the process of EMT induced by PM_2.5_ and NNK (Fig. 2a and b). We found that PM_2.5_ and NNK increased the expression of β-catenin, pGSK3β and TCF4 by either a shorter or longer term of treatment (Fig. 2c and d), indicating that PM_2.5_ could activate Wnt/β-catenin signal pathway in a fashion similar to NNK [24]. We screened some other human lung cell lines such as A549 and NCI-H460, which exhibited some similar results (Additional file 1: Figure S1E). Immune-staining also showed that the membrane vimentin was increased in PM_2.5_- or NNK-treated Bet1A lung cells (Fig. 2e).

#### Downregulation of 15-LOX1/15-LOX2 was correlated with some clinicopathological characteristics regardless of smoking status

**Fig. 3:**
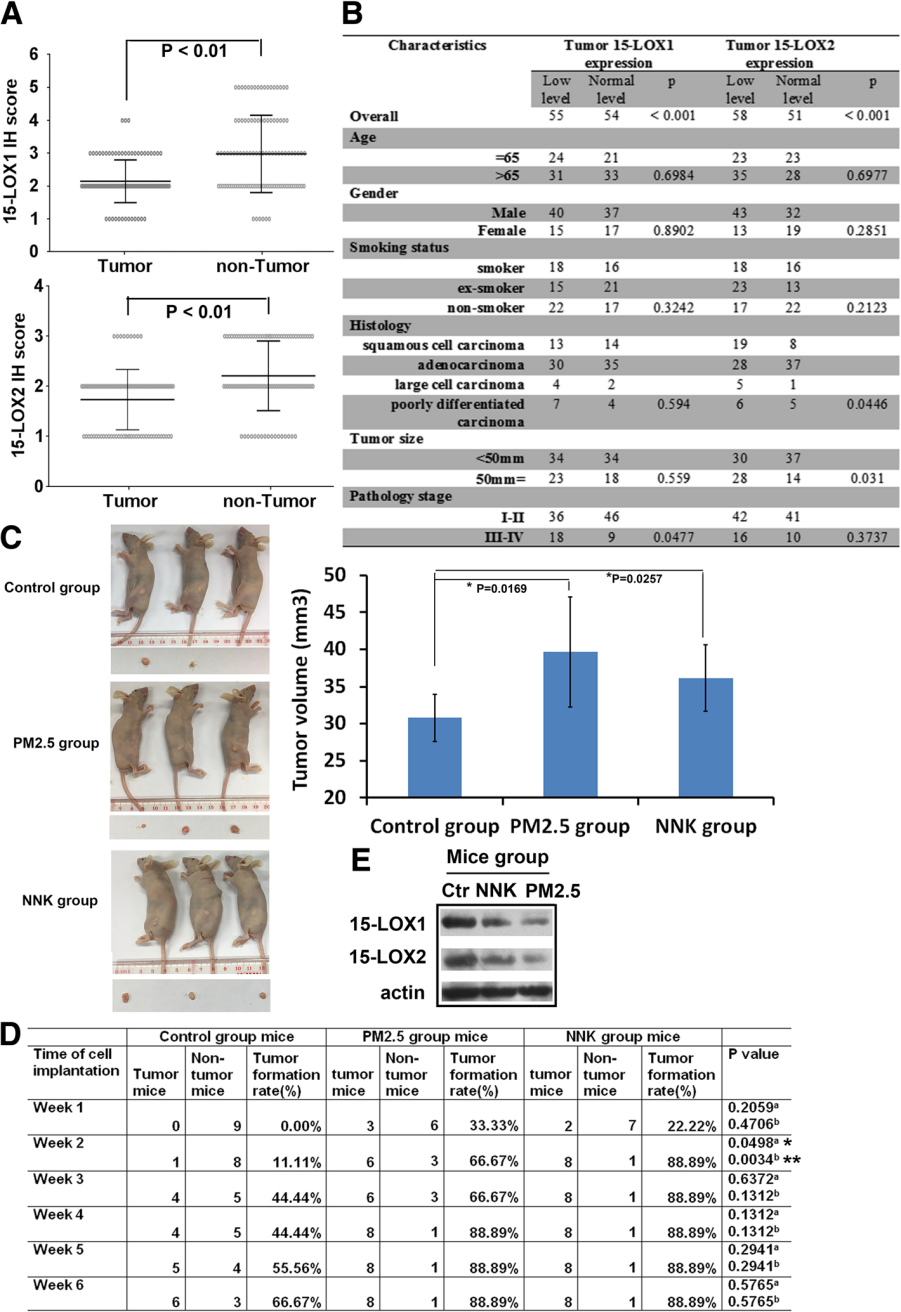
The decreased expression of 15-LOX1 and 15-LOX2 in lung tumor tissues. **a** The levels of 15-LOX1 and 15-LOX2 in 109 NSCLC tissues and paired adjacent normal tissues. The stained tissues were examined and expressed as Mean with range. Wilcoxon signed ranks test was used to compare the values (P < 0.01). **b** Baseline demographic characteristics of 109 human NSCLC patients underwent 15-LOX1 and 15-LOX2 analysis. The clinic-pathologic features in patients with relative expressing 15-LOX1/15-LOX2 were compared using Pearson’s chi-squared test or Fisher’s exact test for categorical variables. P < 0.05 was considered statistically significant. **c** Tumorigenicity assay. The nude mice were transplanted with NCI-H23 cells treated with PM2.5 or NNK for 28 days. Representative images of mice and tumor were shown. The growth of tumors was calculated by tumor volume (*P < 0.05). **d** Tumor formation rate assay. Tumor formation rate in each time-point was recorded (*p < 0.05, **p < 0.01; **a** P value as compared each group of PM2.5-treated condition with the control group; **b** P value as compared each group of NNK-treated condition with the control group. Data are mean ± SD). **e** 15-LOX1 and 15-LOX2 expression in the xenografts of NCI-H23 cells. Three of each group of tumor tissue proteins from week 6 mice was pooled together and 15-LOX1/15-LOX2 expression in the xenografts was detected. Equal loading was confirmed by probing with antibodies against actin

Immunohistochemistry (IHC) and immunoreactivity scoring results showed that 15-LOX1 and 15-LOX2 were expressed respectively in 55/109 (50.5%) and 58/109 (53.2%) of NSCLCs and the overall expression of both was significantly downregulated in cancer tissues than in normal adjacent tissues (Fig. 3a-b). These findings were confirmed by western blotting (Additional file 1: Figure S2A). Unlike 15-LOXs, CD133, a well-known marker of lung CSCs, was upregulated in all NSCLC tumor tissues (Additional file 1: Figure S2A). Our correlation analysis showed that there was no significant association of 15-LOX1 levels with age, gender, smoking status, histology, and tumor size. However, the lower level of 15-LOX1 was significantly associated with pathology state (Fig. 3b). The lower level of 15-LOX2 was significantly associated with tumor size and histology but not with other clinicopathological features (Fig. 3b). 15-LOX1 and 15-LOX2 expression was not significantly different in smoker, ex-smoker and non-smoker patients (Fig. 3b), implicating that they may act as tumor suppressors for NSCLCs in both smokers and non-smokers.

In light of our in vitro results, we evaluated the tumorigenesis of PM_2.5_ and NNK in mice. PM_2.5_- or NNK-treated NCI-H23 cells were injected into the left and right flanks of nude mice. Tumor growth was significantly faster in mice injected with PM_2.5_- or NNK-treated cells than those with non-treated control cells (Fig. 3c). The tumor formation rate in the treated groups was higher than the control group (Fig. 3d). Western blot results revealed a significant reduction in the expression of 15-LOX1 and 15-LOX2 proteins in tumors formed by treated cells, compared with that in tumors formed by the non-treated cells (Fig. 3e). These results supported a tumor-promoting role for PM_2.5_ and NNK, likely via reducing 15-LOXs.

### Overexpression of 15-LOX1/15-LOX2 prevented PM_2.5_- or NNK-induced vimentin expression

**Fig. 4:**
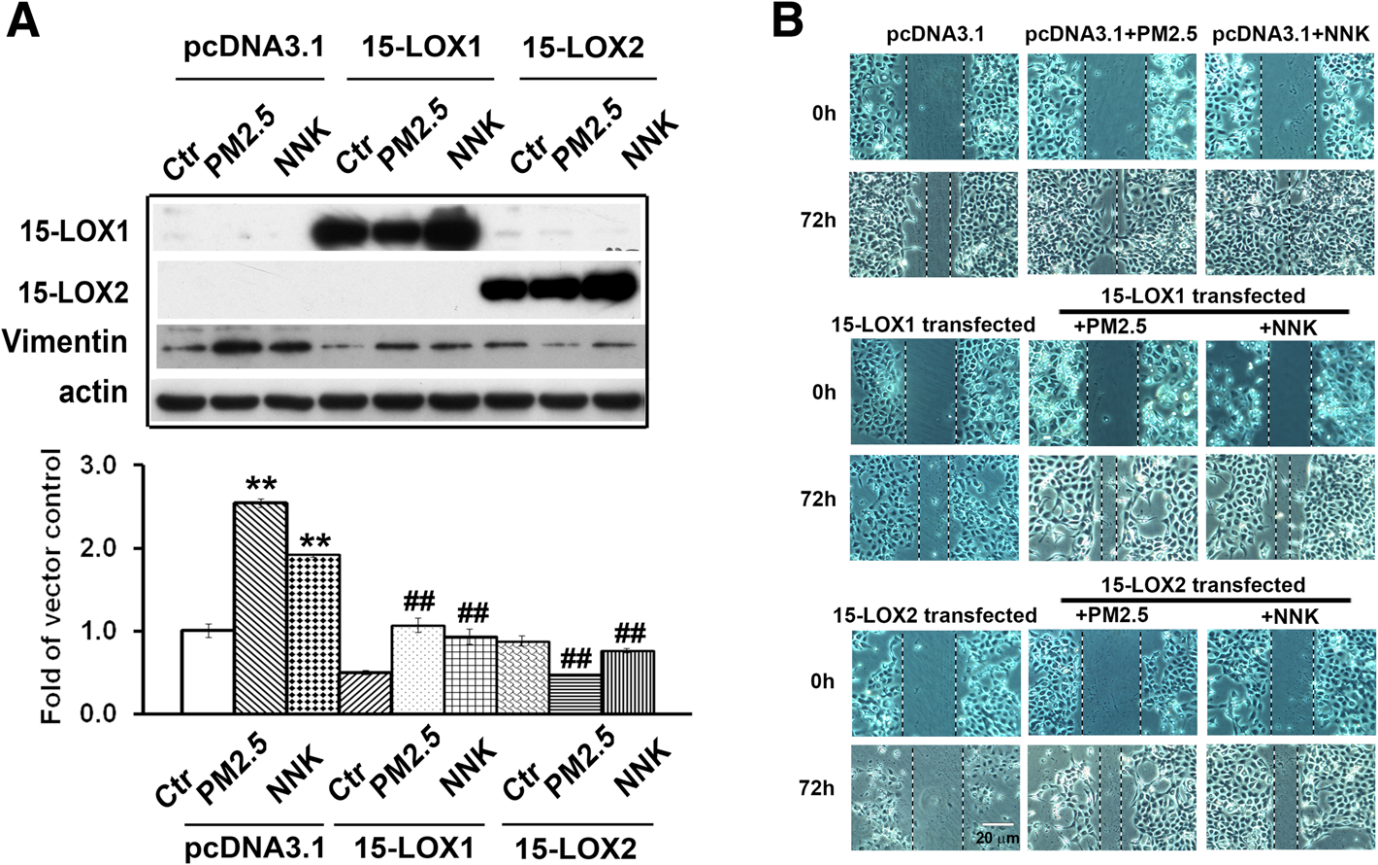
Restoration of 15-LOX1 and 15-LOX2 activities inhibited the effects of PM2.5 and NNK. **a** Restoration of 15-LOX1 and 15-LOX2 activities inhibited the effects of PM2.5 or NNK on vimentin expression. After Bet1A cells were treated with PM2.5 or NNK for 28 days, the cells were transfected with 15-LOX1, 15- LOX2 or vector plasmid DNA respectively for 24 h. 15-LOX1, 15-LOX2 and vimentin were determined. The quantification of protein was carried out by densitometry analysis and expressed as mean ± SE. The relative intensity of protein bands was summarized by column figure (n = 3, **p < 0.01when compared each PM2.5- or NNK-treated condition with the control cells; and ^##^*P* < 0.01 when compared the condition of cells transfected with 15-LOX1/15-LOX2 and treated with PM2.5 or NNK with the condition of cells transfected with pcDNA3.1 and treated with PM2.5 or NNK respectively). **b** Restoration of 15-LOX1 and 15-LOX2 activities inhibited the effects of PM2.5 or NNK on cell migration. Bet1A cells treated with PM2.5 or NNK for 28 days were transfected with 15-LOX1, 15- LOX2 or vector plasmid DNA respectively for 24 h. Wound-healing assay were performed

Vimentin is the well-characterized biomarker of EMT during lung carcinogenesis [26]. From IHC results, Vimentin expression in 68/109 (62.4%) of patients was higher in the tumor samples than their adjacent normal controls. The overall expression of Vimentin was significantly higher in cancer tissues than in adjacent normal tissues (p < 0.01) (Additional file 1: Figure S2B and Table S1). To confirm this finding, western blotting was performed to detect their protein levels in 6 randomly selected paired NSCLC specimens. As shown in Additional file 1: Figure S2C, 4 out of 6 of patients showed higher level of Vimentin protein in tumor tissues than that in adjacent normal tissues. The expression of vimentin was significantly associated with age but not with other clinicopathological features (Additional file 1: Table S1). Importantly, we found that PM_2.5_- or NNK-mediated vimentin expression could be significantly inhibited by 15-LOX1 and 15-LOX2 overexpression (Fig. 4a). Some of other lung carcinogenesis related proteins such as ALDH1A1, TCF4, and Bcl-2 in Bet1A cells induced by PM_2.5_ or NNK were also inhibited by overexpression of 15-LOX1 and 15-LOX2. At the same time, the overexpression of 15-LOX1 and 15-LOX2 promoted procaspase 3 into its cleaved active format (Additional file 1: Figure S2D). The overexpression of 15-LOX1 and 15-LOX2 were also significantly reduced the cell migration (Fig. 4b and Additional file 1: Figure S2E).

### Expression of 15-LOX1 was epigenetically regulated at methylation level by PM_2.5_ and NNK

**Fig. 5:**
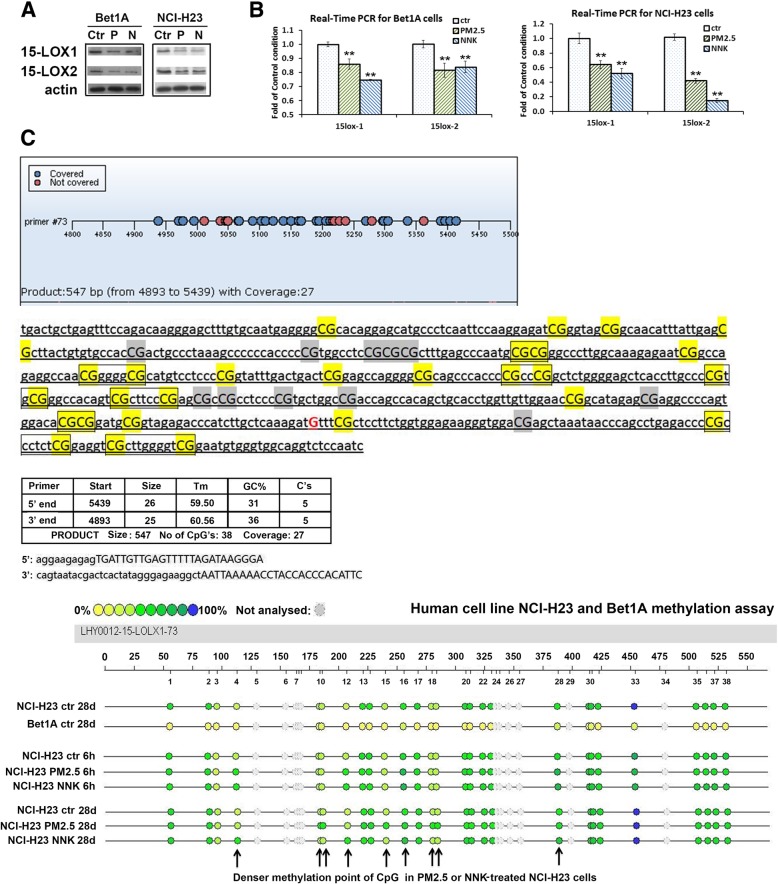
15-LOX1 is partially silenced by promoter methylation in human lung carcinogenic cells. **a** and (**b**) Downregulation of 15-LOX1 and 15-LOX2 by PM2.5 or NNK. Bet1A and NCI-H23 cells were treated with PM2.5 or NNK for 28 days. Total protein and RNA were extracted for (**a**) Western-blot and (**b**) real-time PCR assay (n = 3, **p < 0.01when compared each PM2.5- or NNK-treated condition with the control cells). **c** MassArray for methylation assay. One CpG island was found in 15-LOX1 promotor region. CpGs within the 15-LOX1 locus were hypermethylated in lung cancer cell NCI-H23 as compared with normal lung epithelial cell Bet1A. More denser methylation was also observed in some points of PM2.5 and NNK-treated NCI-H23 cells than NCI-H23 control cells as indicated with the arrow

To explore the mechanism that PM_2.5_ and NNK downregulated the expression of 15-LOX1 and 15-LOX2 (Fig. 5a and b), MassArray for methylation assay (BGI, china) of genes was employed to detect the 15-LOX1/15-LOX2 gene promotor methylation level. The methylation level of 15-LOX1 gene was much higher in lung cancer cell line NCI-H23 than that in the normal lung epithelial cell Bet1A (Fig. 5c and Additional file 1: Table S2), indicating that the low expression of 15-LOX1 gene in lung cancer was at least partially due to the methylation regulation. In NCI-H23, the 6-h short-period and 28-day long-period treatments with PM_2.5_ or NNK partially enhanced methylation of some of CpG islands of 15-LOX1 gene (Fig. 5c and Additional file 1: Table S2). However, 15-LOX1 gene exhibited low methylation level in Bet1A lung epithelial cells and we could not see the significant change of methylation level after the cells treated with PM_2.5_ or NNK (Additional file 1: Table S2). The negative results might be due to the fact that the treatment period was not long enough to induce more malignant transformation of Bet1A epithelial cells or other unknown factors.

### Expression of 15-LOXs was post-transcriptionally regulated by microRNAs

**Fig. 6:**
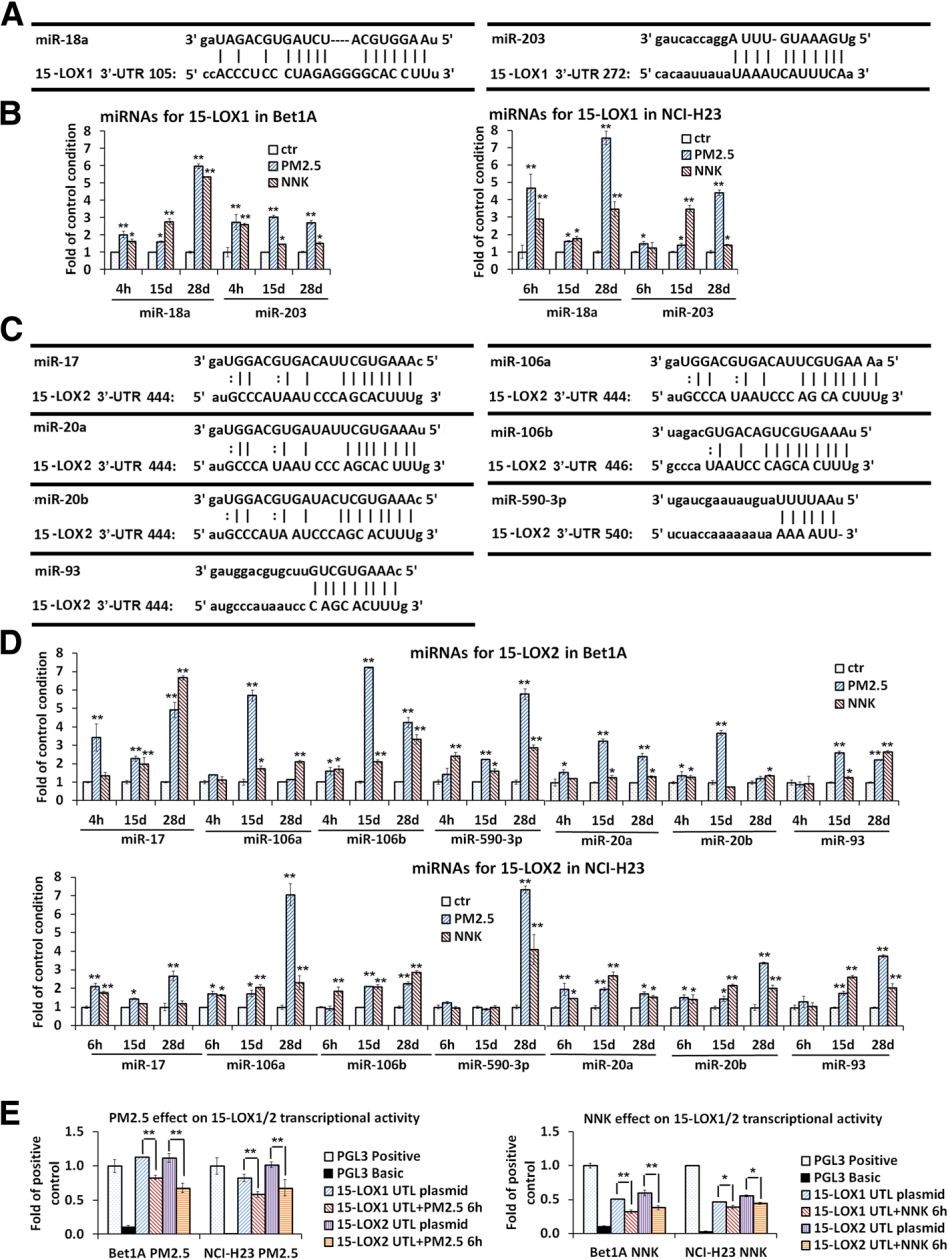
miRNAs downregulated the expression of 15-LOX1 and 15-LOX2 in PM2.5- or NNK-induced lung carcinogenic cells. Predicted binding sites of miRNAs on 15-LOX1 3′-UTR region (**a**); and on 15-LOX2 3′-UTR region (**c**). The alignment of miR-18a, miR-203 with two predicted binding sites of 15-LOX1 3′-UTR were shown; The alignment of miR-17, miR-20a, miR-20b, miR-93, miR-106a, miR-106b, and miR590-3p with three predicted binding sites of 15-LOX2 3′-UTR were shown. **b** miRNAs targeted on 15-LOX1 3′-UTR region were upregulated; and (**d**) miRNAs targeted on 15-LOX2 3′-UTR region were upregulated in PM2.5- or NNK-induced lung carcinogenic cells. Bet1A and NCI-H23 cells were treated with PM2.5 or NNK for 4 h, 15 days, and 28 days. Total RNA were extracted for real-time PCR assay (*n* = 3, *p < 0.05 and **p < 0.01when compared each PM2.5 or NNK treated condition with the control cells.). **e** 15-LOX1 and 15-LOX2 transcriptional activity was downregulated by PM2.5 or NNK. Bet1A or NCI-H23 cells were transfected with luciferase reporter constructs, pGL3–15-LOX1 3′-UTR or pGL3–15-LOX2 3′-UTR for 24 h, the pGL3 basic vector and the pGL3 control were used as negative and positive controls, respectively, followed by PM2.5 or NNK treatment for 6 h. Reporter assays were performed using the Dual-luciferase assay system, normalized for transfection efficiency by co-transfected Renilla luciferase (*n* = 3, *p < 0.05 and **P < 0.01, compared with the cells transfected with 15-LOX1/2 3′-UTR alone)

Real-time PCR results showed that several miRNAs that could target 15-LOX1 or 15-LOX2 3′-UTR region were significantly increased by PM_2.5_ or NNK treatment (Fig. 6a-d). miR-18a and miR-203 which targeted two sites of 3′-UTR of 15-LOX1 were upregulated (Fig. 6a-b). Seven miRNAs miR-17, miR-20a, miR-20b, miR-106a, miR-106b, miR-93, and miR-590-3p which targeted three sites of 3′-UTR of 15-LOX2 were increased by PM_2.5_ or NNK treatment (Fig. 6c-d). In consistence with these results, the activities of 15-LOX1 3′-UTR reporter and 15-LOX2 3′-UTR reporter were significantly downregulated in PM_2.5_- or NNK-treated lung cells compared with that in the control cells (Fig. 6e), suggesting that these nine miRNAs tested here could function as the multiple negative regulators of 15-LOXs.

## Discussion

The acquisition of cellular characteristics of lung malignancy is critically linked to unregulated cell proliferation, formation of CSC, and EMT alterations, resulting in tumor growth, invasion and metastasis. Long-term cell cultures have been used to study lung tumorigenesis. For example, inflammatory factors critical for carcinogenesis were upregulated by a long period (60 days) treatment with NNK in normal human lung epithelial cells 16HBE cells [27]. Compared with the short-term exposure to NNK, long-term NNK treatment accompanied with the increased resistance to cisplatin-induced apoptosis of lung epithelial or lung cancer cells [28]. In the malignant transformation of normal human bronchial epithelial cells induced by NNK, the expression of Survivin protein was markedly increased by either short-term (12 h to 3 days) or long-term (90 days) treatment with NNK [29]. NNK enhanced the proliferation of BEAS-2B normal lung epithelial cells through DNA methylation up to 5 weeks after NNK treatment [30]. In our present study, we treated cells with PM_2.5_ or NNK for both a short-term period and a long-term period. This study demonstrated that not only NNK treatment but also PM_2.5_ treatment could increase lung cell carcinogenesis property including the enhancement of cell proliferation, CSC formation, and cell invasion and migration. Wnt/β-catenin signal pathway was involved in both NNK- and PM_2.5_-induced lung carcinogenesis. The results clearly showed that PM_2.5_ could promote lung cell carcinogenesis property in the way very similar to the smoking carcinogen NNK, providing molecular evidence to support PM_2.5_, like NNK, as a powerful oncogenic factor in lung. Tumor-inducing effects of PM_2.5_ and NNK were validated in vivo, as evidenced by the enhanced growth of PM_2.5_- or NNK-treated NCI-H23 cells in nude mice. The in vivo data obtained here confirmed the tumorigenesis effect of PM_2.5_- as well as NNK-treated NCI-H23 cells. Although NCI-H23 cells themselves are able to form tumors in mice [31], PM_2.5_ or NNK can significantly facilitate and speed up this process, which was consistent with previous studies that showed a significant positive correlation between PM2.5 concentration and lung cancer mortality, and PM_2.5_ exposures after lung cancer diagnosis shorten survival [1, 7]. Bet1A cells maintain a non-transformed phenotype, and thus they will not be able to form tumors in mice. We hypothesized that PM_2.5_ or NNK could change the phenotype of Bet1A cells so that they were able to form tumors in vivo. We observed tumor-like lumps in Week 1 and 2 after the implantation of PM_2.5_ or NNK-treated Bet1A cells in mice. However, these lumps disappeared afterwards. According to our results for the self-renewable ability of the CSCs, it showed that the NCI-H23 CSCs exhibited much more vigorous self-development than that of Bet1A CSCs. Thus, the failure of the tumorigenesis of PM_2.5_- or NNK-treated Bet1A cells indicates that 28-days of PM_2.5_ or NNK treatment are not long enough to make these CSC-like Bet1A cells tumorigenically stable enough to generate tumors. It will be worth trying a longer period of cell cultures to test tumorigenic effects of PM.5 and NNK on normal lung epithelial cells.

The metallic chemical components of PM_2.5_ in the sampling site well followed the profile of other sites while their concentrations varied [17]. In Guangzhou and Beijing, almost all the concentrations of components showed two orders higher in magnitude than those in Vienna [17]. It seemed that the concentration of PM_2.5_ played a dominant role in inducing lung cancer, which was consistent with the evidence that the incidence of LCNS is increasing in some large Chinese cities with heavily polluted air [2]. Increased risk estimates of lung cancer were observed for each 10 μg/m^3^ increment in ambient PM_2.5_ concentration [32]. Similarly, it was reported a 37% stronger association with lung cancer for each 10 μg/m^3^ increment in PM_2.5_ [33]. A significant positive correlation between PM2.5 concentration and lung cancer mortality was found [1]. The molecular data on the PM_2.5_-induction of lung cell carcinogenesis reported here, though are original, should be deemed incompleted. Potential biomarkers identified should be further studied for possible targeting therapies and the early diagnosis of lung cancer, particularly for PM_2.5_-induced LCNS in the areas with severe air pollution.

Our previous finding suggested that 15-LOXs functioned as a tumor suppressor in NSCLC [14, 15], this result is further supported by current data showing a positive relationship between downregulation of 15-LOXs and the lung carcinogenesis, which was regardless of the smoking status. Epigenetic regulation plays a critical role in repressing gene expression and maintaining genomic stability [34]. A recent analysis identified 66 differentially expressed genes in PM2.5-induced human alveolar epithelial cells, which were either hyper- or hypo-methylated, particularly involved in lung cancer [35]. In this study, MassArray for methylation assay found one CpG island in 15-LOX1 promotor region, while no CpG island was predicted in 15-LOX2 gene. The methylation level of 15-LOX1 gene was much higher in lung cancer cell NCI-H23 than that in the lung epithelial cell Bet1A, indicating that the low expression of 15-LOX1 gene in lung cancer was at least partially due to the methylation regulation. PM_2.5_ or NNK treatment partially enhanced methylation of some of CpG islands of 15-LOX1 gene in NCI-H23, but not in Bet1A lung epithelial cells (Fig. 5c and Additional file 1: Table S2). Moreover, our luciferase assay of lung cells transfected with 3′-UTR of 15-LOX1 and 15-LOX2 plasmids have demonstrated that 15-LOX1 and 15-LOX2 expression could be significantly inhibited post-translationally by microRNAs (Fig. 6). MicroRNAs can negatively regulate gene expression by binding to 3′-UTR of target mRNAs; causing translational repression or degradation of target mRNAs [36]. Human bronchial epithelial cells exposed to PM2.5 showed the downregulation of miR-182 and miR-185 expression, resulting in their three target oncogenes were markedly induced to cause neoplastic transformation [37]. Our real-time PCR results showed that miR-18a and miR-203 that can potentially bind to four sites of 3′-UTR of 15-LOX1, and miR-17, miR-20a, miR-20b, miR-93, miR-106a, miR-106b and miR-590-3p that can potentially bind to three sites of 3′-UTR of 15-LOX2 were induced by PM_2.5_ or NNK. Among these 9 detected miRNAs, miR-17, miR-18a and miR-20a belonged to the miR-17/92 cluster which was known as ‘oncogene’, and miR-106a, miR-20b, miR-106b, and miR-93 belonged to miR17 family [38, 39]. These miR-17/92 cluster microRNAs could promote the high proliferation and undifferentiated phenotype of lung epithelial progenitor cells and were overexpressed in solid cancers including lung cancer [38, 40]. These results have demonstrated that PM_2.5_ and NNK downregulate the expression of 15-LOXs via multiple pathways.

## Conclusions

In summary, PM2.5 can function as the smoking carcinogen NNK to induce lung tumorigenesis by inhibiting 15-LOX1/15-LOX2. The findings have potential implications in clinical management of patients with NSCLC, as molecules identified may offer new biomarkers for early diagnosis of lung cancer, new targets for the treatment of NSCLC, particularly for PM_2.5_-related LCNS. The emerging role of microRNA-regulated gene networks and biological pathways associated with lung cancer may provide new hope for the identification of non-invasive and accurate microRNA signatures to early diagnosis, and guide therapeutic decisions for lung cancer patients. The result generated also sends an urgent message to the public to take more effective actions to control the air pollution.

## Additional file

Additional file 1: **Figure S1.** (A) PM2.5 and NNK induced lung Bet1A1 and NCI-H23 cell proliferation. (B) Sphere formation of lung cancer stem cells induced by PM2.5 or NNK. (C) CSC tumor sphere assay on PM2.5- or NNK-treated Bet1A and NCI-H23 cells. (D) PM2.5 induced Bet1A and NCI-H23 cell invasion. (E) PM2.5and NNK treatment with 28 days promoted the expression of lung cell carcinogenesis-related biomarkers. **Figure S2.** (A) 15-LOX1 and 15-LOX2 expression in human lung tumor tissues and non-tumor tissues. (B) The levels of vimentin in 109 paired NSCLC tissues and adjacent normal non-tumor tissues. (C) Vimentin expression in human lung tumor tissues and non-tumor tissues. (D) Restoration of 15-LOX1 and 15-LOX2 activities inhibited the effects of PM2.5 or NNK on the expression of lung carcinogenetic proteins. (E) Restoration of 15-LOX1 and 15-LOX2 activities inhibited the effects of PM2.5 or NNK on cell migration. **Figure S3.** MassArray design for 15-LOX1 methylation detection. (A) Sequence information of 15-LOX1 methylation design. (B) Prediction of potential CpG islands using http://www.ebi.ac.uk/Tools/seqstats/emboss_cpgplot/ website. (C) Primers design using sequenom®EpiDesigner program. **Figure S4.** Cloning of 15-LOX1 3'-UTR and 15-LOX2 3'-UTR. Table S1. Baseline demographic characteristics of 109 human NSCLC patients underwent Vimentin analysis. Table S2. Human 15-LOX1 gene methylation level in NCI-H23 and Bet1A cells treated by PM2.5 and NNK. (DOCX 8597 kb)

## Data Availability

All relevant data are included in the paper and its supplementary information files.

## Abbreviations

15-LOXs: 15-lipoxygenases
CSC: Cancer stem cell
EMT: Epithelial mesenchymal transition
LCNS: Lung cancer in never smokers
NNK: 4-methylnitrosamino-l-3-pyridyl-butanone
NSCLCs: Non-small cell lung cancers
PM_2.5_: Atmospheric particulate matter with *d*_p_ < 2.5 μm
