# Supplementary material for: Ambient fine particulate matter inhibits 15-lipoxygenases to promote lung carcinogenesis
Source: J Exp Clin Cancer Res. 2019 Aug 16;38:359. doi: 10.1186/s13046-019-1380-z (PMC6697918; doi:10.1186/s13046-019-1380-z)
Supplement: Supplementary file 1 — Figure S1. (A) PM2.5 and NNK induced lung Bet1A1 and NCI-H23 cell proliferation. (B) Sphere formation of lung cancer stem cells induced by PM2.5 or NNK. (C) CSC tumor sphere assay on PM2.5- or NNK-treated Bet1A and NCI-H23 cells. (D) PM2.5 induced Bet1A and NCI-H23 cell invasion. (E) PM2.5and NNK treatment with 28 days promoted the expression of lung cell carcinogenesis-related biomarkers. Figure S2. (A) 15-LOX1 and 15-LOX2 expression in human lung tumor tissues and non-tumor tissues. (B) The levels of vimentin in 109 paired NSCLC tissues and adjacent normal non-tumor tissues. (C) Vimentin expression in human lung tumor tissues and non-tumor tissues. (D) Restoration of 15-LOX1 and 15-LOX2 activities inhibited the effects of PM2.5 or NNK on the expression of lung carcinogenetic proteins. (E) Restoration of 15-LOX1 and 15-LOX2 activities inhibited the effects of PM2.5 or NNK on cell migration. Figure S3. MassArray design for 15-LOX1 methylation detection. (A) Sequence information of 15-LOX1 methylation design. (B) Prediction of potential CpG islands using http://www.ebi.ac.uk/Tools/seqstats/emboss_cpgplot/ website. (C) Primers design using sequenom®EpiDesigner program. Figure S4. Cloning of 15-LOX1 3'-UTR and 15-LOX2 3'-UTR. Table S1. Baseline demographic characteristics of 109 human NSCLC patients underwent Vimentin analysis. Table S2. Human 15-LOX1 gene methylation level in NCI-H23 and Bet1A cells treated by PM2.5 and NNK. (DOCX 8597 kb) [file 13046_2019_1380_MOESM1_ESM.docx]

Supplementary Materials

**Ambient fine particulate matter inhibits 15-lipoxygenases to promote lung carcinogenesis**

Ming-Yue Li^1,3#*^, Li-Zhong Liu^2#^, Wende Li^6^, Calvin SH Ng^1^, Yi Liu^1,7^, Angel WY Kong^1^, Zhili Zhao^1^, Shanshan Wang^10^, Haolong Qi^1^, Hao Jia^1^, Shucai Yang^8^, Jing Du^9^, Xiang Long^9^, Rocky L.K. Ho^1^, Ernest C. W. Chak^1^, Innes YP Wan^1^, Tony SK Mok^5^, Malcolm J Underwood^1^, Nirmal Kumar Gali^4^, Zhi Ning^4,*^, George G Chen^1,3*^

**#These authors contributed equally to this work.**

^1^Department of Surgery, ^5^Department of Clinical Oncology, and ^10^Department of Otorhinolaryngology, Head and Neck Surgery, The Chinese University of Hong Kong, Prince of Wales Hospital, Shatin N.T., Hong Kong.

^2^Faculty of Medicine, Shenzhen University Health Science Center, Shenzhen University, Shenzhen, China.

^3^Shenzhen Research Institute, the Chinese University of Hong Kong, Shenzhen, Guangdong, China.

^4^Division of Environment and Sustainability, The Hong Kong University of Science and Technology, Hong Kong.

^6^Guangdong Key laboratory of Laboratory Animal, Guangdong Laboratory Animals Monitoring Institute, Guangzhou, China

^7^Guangdong Medical College, Zhangjiang, Guangdong, China

^8^Department of Clinical Laboratory, Pingshan District People's Hospital Of Shenzhen, Shenzhen, China

^9^Peking University Shenzhen Hospital, Shenzhen, Guangdong, China.

**
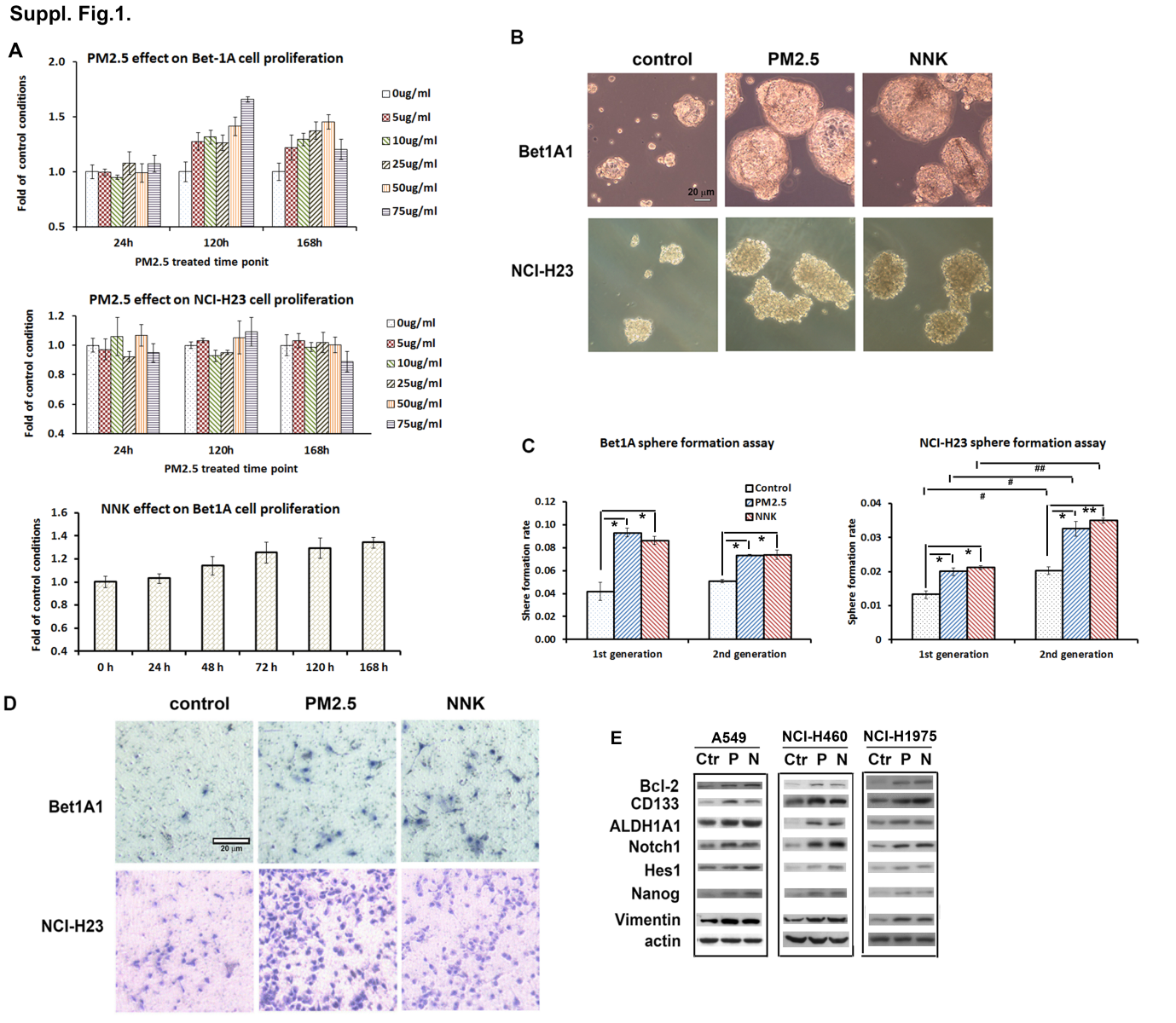
Additional files**

**Figure S1. (A) PM_2.5_ and NNK induced lung Bet1A1 and NCI-H23 cell proliferation.** Cells were treated with different concentrations of PM_2.5_ for 24h, 120h and 168h respectively or treated with 10 µM NNK for 24h, 48h, 72h, 120h and 168h respectively, and measured by MTT assay. (∗*P <* 0*.*05, ** *P < 0.01, n* = 4). **(B)** **Sphere formation of lung cancer stem cells induced by PM_2.5_ or NNK.** Cells were treated with PM_2.5_ or NNK for 6h. No treatment cells were set up as the control. The cells were dissociated into single-cell suspension and plated in ultra-low adhesion 6-well plates at the density of 2500 cells/well in cancer stem cell medium to grow for 10 days. Images of the spheres were taken using phase contrast microscope (Nikon) (original magnification ×200, scale bar, 20 μm). **(C) CSC tumor sphere assay on PM_2.5_- or NNK-treated Bet1A and NCI-H23 cells.** Cells were treated with PM_2.5_ or NNK for 6h. No treatment cells were set up as the control. The cells were dissociated into single-cell suspension and plated in ultra-low adhesion 6-well plates at the density of 2500 cells/well in cancer stem cell medium to grow for 10 days. Total number and size of spheres more than 60$\mu$m were counted. The first-generation tumor sphere cells generated were dissociated into single-cell suspension by Cell Dissociation Reagent. Cells were cultured in Cancer Stem PremiumTM media to obtain second-generation spheres. Tumor spheres were counted to study the self-renewal of CSCs. (n=3, *p<0.05 and **P<0.01, compared the cells treated with PM2.5 or NNK with control cells; ^#^p<0.05 and ^##^P<0.01, compared the relative cells of the 1^st^ generation with 2^nd^ generation.). **(D) PM_2.5_ induced Bet1A and NCI-H23 cell invasion**. Cells were treated by PM_2.5_ or NNK for 28 days. Then the cells were seeded for different periods. Images were taken using phase contrast microscope (Nikon) (scale bar, 20 μm). **(E)** **PM_2.5_ and NNK treatment with 28 days promoted the expression of lung cell carcinogenesis-related biomarkers.** NCI-H460, A549 and NCI-H1975 purchased from the American Type Culture Collection (ATCC) were cultured in RPMI-1640 medium, supplemented with 10% inactivated FBS. A549, NCI-H460 or NCI-H1975 cells were treated with 5µg/ml PM_2.5_ or 10 µM NNK for 28 days. Non-treated cells were cultured for 28 days as the control condition. Lung carcinogenesis-related biomarkers Bcl-2, CD133, ALDH1A1, Notch1, Hes1, Nanog, and vimentin were detected by western blot. The equal loading was confirmed by measuring actin protein.

**
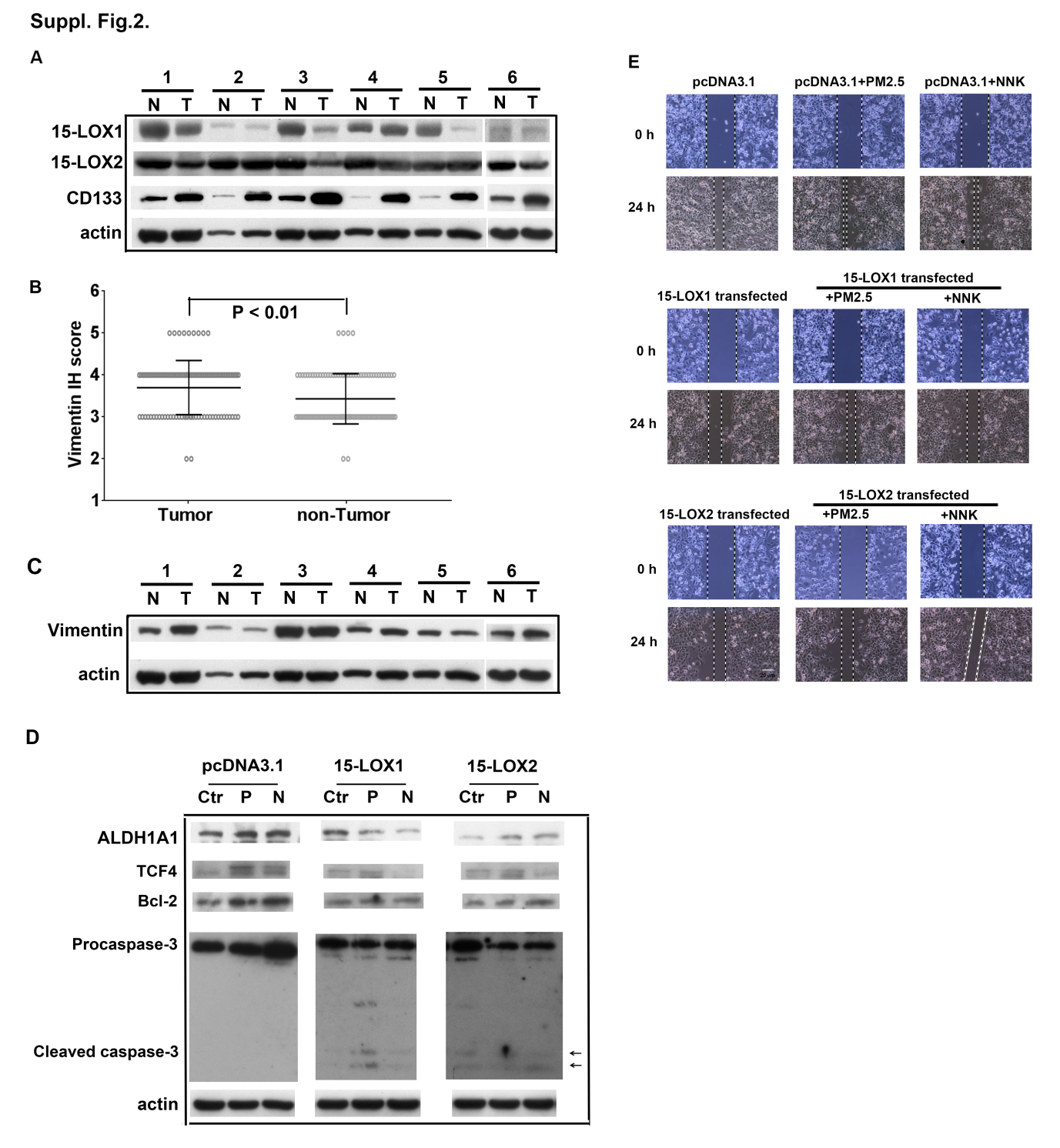
**

**Figure S2. (A) 15-LOX1 and 15-LOX2 expression in human lung tumor tissues and non-tumor tissues.** Tissue proteins were isolated for Western-blot to detect 15-LOX1, 15-LOX2, and CD133 protein levels in 6 randomly selected paired NSCLC specimens. Equal loading was confirmed by probing with antibodies against actin. 3 out of 6 and 4 out of 6 of patients showed lower levels of 15-LOX1 and 15-LOX2 proteins respectively in tumor tissues than that in adjacent normal non-tumor tissues. CD133, as the widely used marker of lung CSCs, was upregulated in all NSCLC tumor tissues. The experiments were repeated twice and similar results were obtained. (N stands for non-tumor tissue, T stands for tumor tissue). **(B) The levels of vimentin in 109 paired NSCLC tissues and adjacent normal non-tumor tissues.** The stained tissues were examined using the Zeiss Spot imaging system and the immunohistochemical staining was assessed and expressed as Mean with range. Wilcoxon signed ranks test was used to compare the values between tumor tissues and non-tumor tissues. **(C)** V**imentin expression in human lung tumor tissues and non-tumor tissues.** Total proteins were isolated for Western-blot. Equal loading was confirmed by probing with antibodies against actin. The experiments were repeated twice and similar results were obtained (N stands for protein form non-tumor tissue; T stands for protein form tumor tissue.) **(D)** **Restoration of 15-LOX1 and 15-LOX2 activities inhibited the effects of PM_2.5_ or NNK on the expression of lung carcinogenetic proteins.** Bet1A cells were treated with PM_2.5_ or NNK for 28 days. After the cells were transfected with 15-LOX1, 15- LOX2 or vector plasmid DNA respectively, cells were incubated for 24 h. The levels of 15-LOX1, 15-LOX2 and ALDH1A1, TCF4, Bcl-2 and Caspase 3 were determined by Western-blot. The equal loading was confirmed by measuring β-tubulin protein (Ctr: non-treatment control; P: PM_2.5_; N: NNK). **(E) Restoration of 15-LOX1 and 15-LOX2 activities inhibited the effects of PM2.5 or NNK on cell migration.** NCI-H23 cells were treated with PM_2.5_ or NNK for 28 days. Then the cells were seeded onto 6-well plates and transfected with 15-LOX1, 15- LOX2 or vector plasmid DNA respectively for 24 h. Wound-healing assay were then performed for cell migration detection.

**
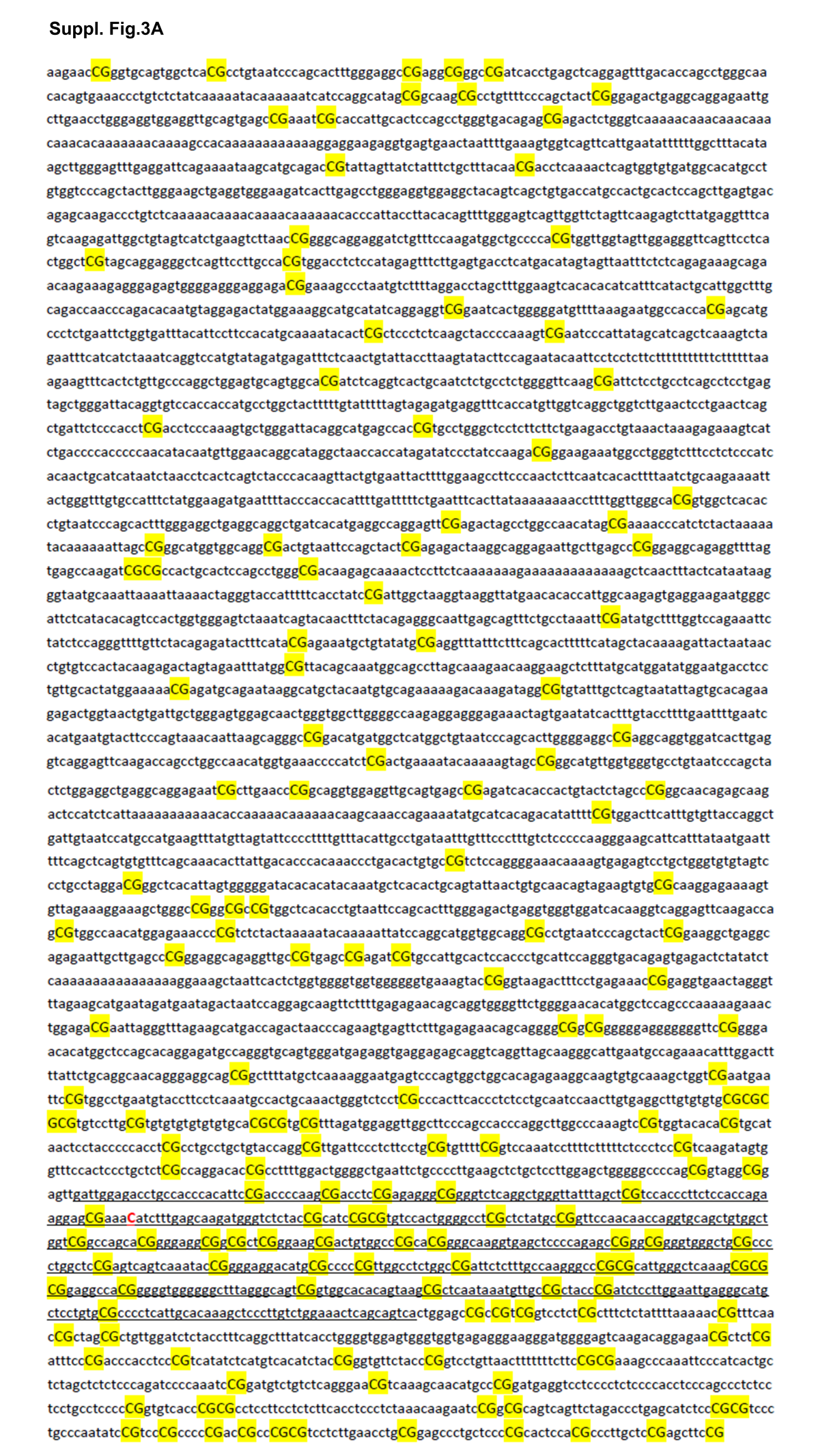
**

**
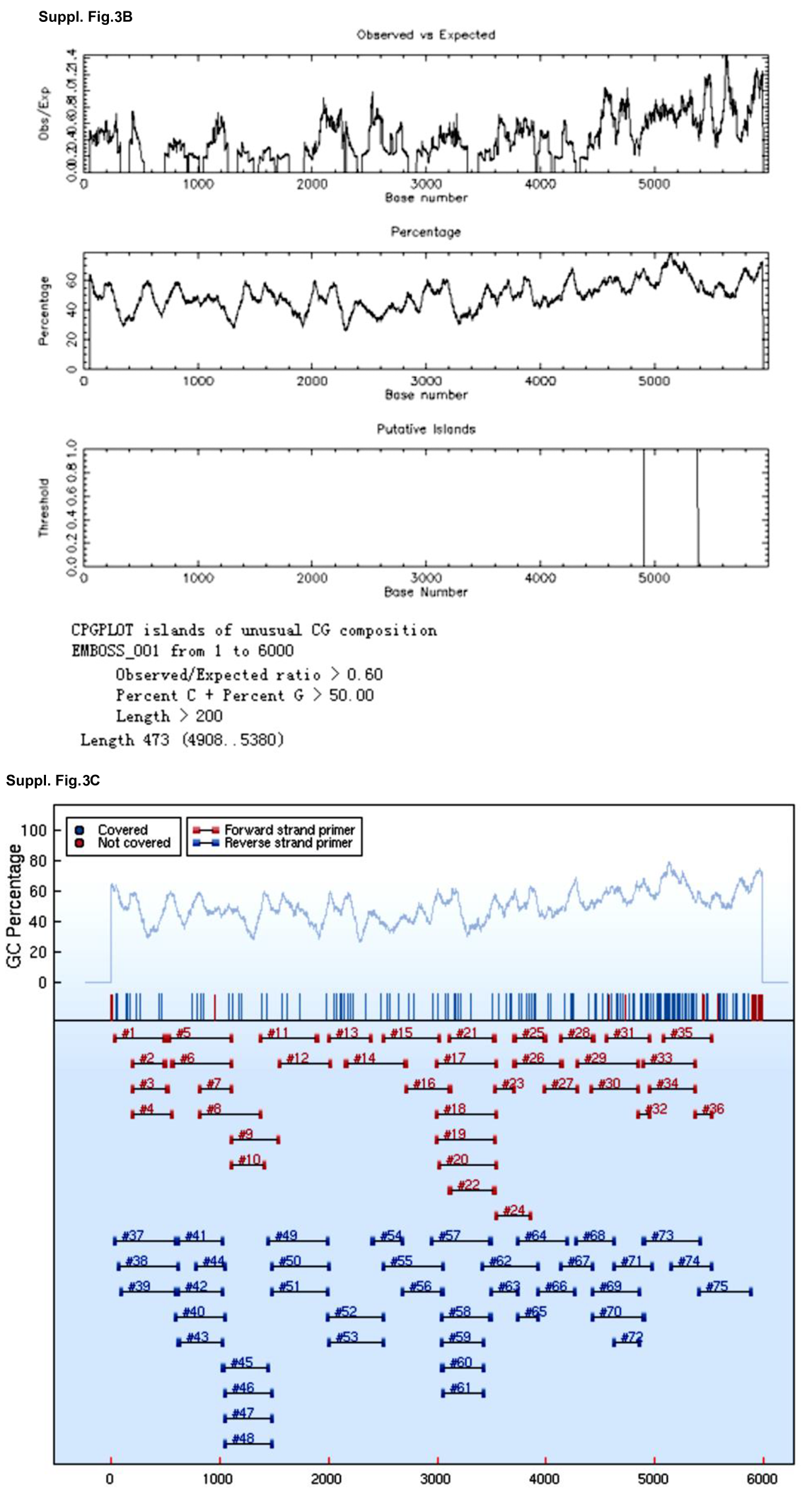
**

**Figure S3. MassArray design for 15-LOX1 methylation detection. (A) Sequence information of 15-LOX1 methylation design.** The sequence site is located at 4640666-4646665 of Chromosome 17 (<https://www.ncbi.nlm.nih.gov/gene/246>). C is the transcription site (TSS) and this 6000 bp sequence is a span of -5000~ +1000 bp of TSS site. **(B)** **Prediction of potential CpG islands using** [**http://www.ebi.ac.uk/Tools/seqstats/emboss_cpgplot/**](http://www.ebi.ac.uk/Tools/seqstats/emboss_cpgplot/) **website.** One potential CpG island was found which located from 4908-5380 bp (Length 473 bp) of the 6000 bp sequence analyzed. **(C) Primers design using sequenom®EpiDesigner program.** Plan #73 was recommended and selected for the methylation assay.


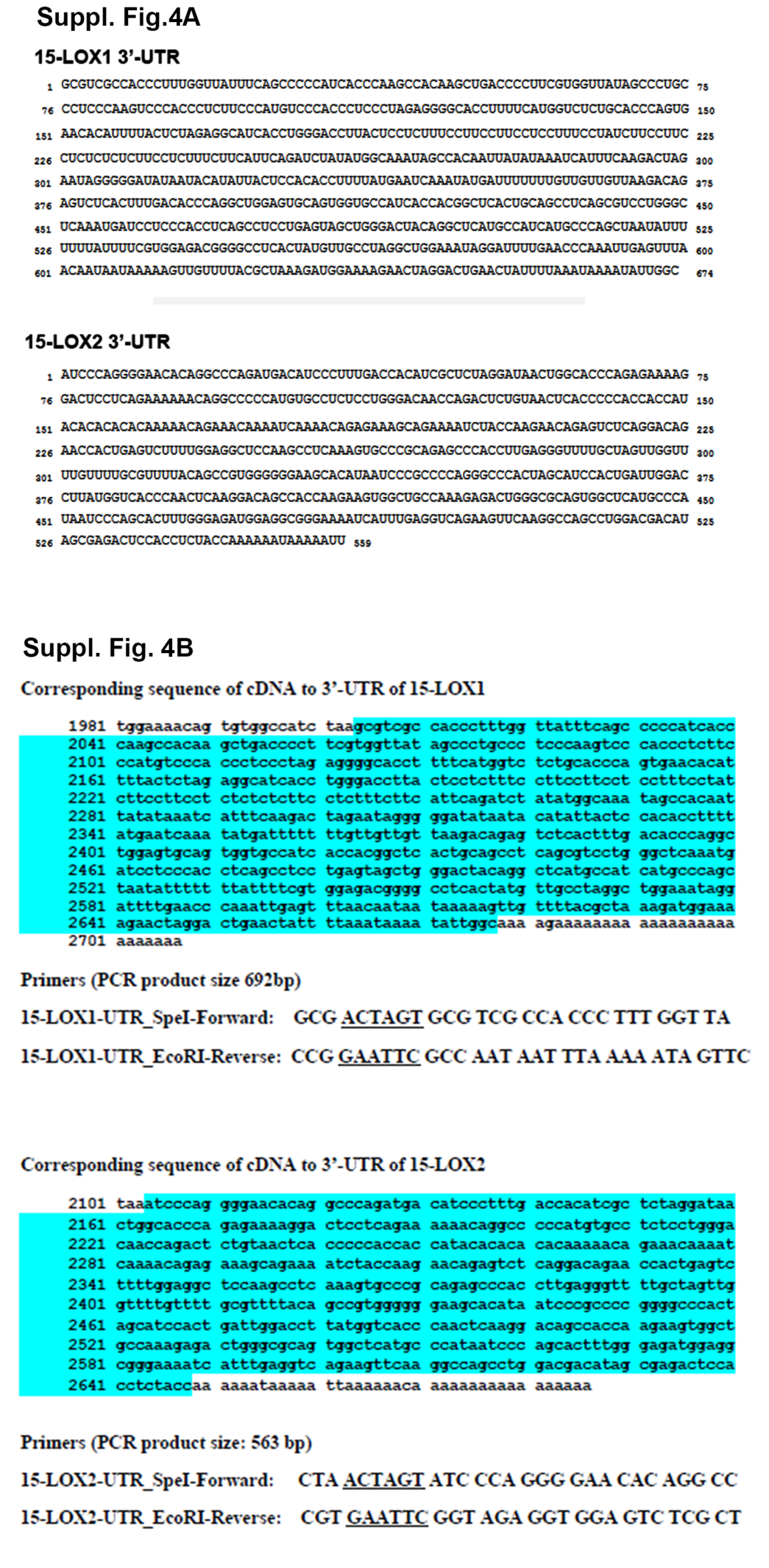


**Figure S4. Cloning of 15-LOX1 3’-UTR and 15-LOX2 3’-UTR.** The Luc-15LOX1/2wt with full-length 3’-untranslated region (UTR) of 15-LOX1 (674 nt) and 15-LOX2 (545 nt) were cloned into pGL3-promoter-vector. **(A) The sequence of 15-LOX1 3’-URT and 15-LOX2 3’-URT.** **(B)** **Cloning of 15-LOX1 3’-UTR and 15-LOX2 3’-UTR** **into the pGL3-promoter-vector.** The cDNA fragment of 15-LOX1 as <http://www.ncbi.nlm.nih.gov/nuccore/20809660> (2004-2677) was amplified by reverse-transcription PCR using the following primer: **15-**LOX1-UTR_SpeI-Forward GCGACTAGTGCGTCGCCACCCTTTGGTTA and 15-LOX1-UTR_EcoRI-Reverse CCGGAATTCGCCAATATTTTATTTAAAATAGTTC; The cDNA fragment of 15-LOX2 <http://www.ncbi.nlm.nih.gov/nuccore/BC063647.1> (2104-2648) was amplified by reverse-transcription PCR using the following primers: 15-LOX2-UTR_SpeI-Forward CTAACTAGTATCCCAGGGGAACACAGGCC and 15-LOX2-UTR_EcoRI-Reverse CGTGAATTCGGTAGAGGTGGAGTCTCGCT. The PCR products were 692bp and 563bp respectively.

**Table S1. Baseline demographic characteristics of 109 human NSCLC patients underwent Vimentin analysis.**

| Characteristics | Tumor Vementin expression | | |
| --- | --- | --- | --- |
|  | High level | Normal level | p |
| Overall | 61 | 48 | < 0.001 |
| Age |  |  |  |
| ≤65 | 30 | 14 |  |
| >65 | 31 | 34 | 0.0345 |
| Gender |  |  |  |
| Male | 42 | 35 |  |
| Female | 19 | 13 | 0.8985 |
| Smoking status |  |  |  |
| smoker | 17 | 17 |  |
| ex-smoker | 18 | 18 |  |
| non-smoker | 26 | 13 | 0.2438 |
| Histology |  |  |  |
| squamous cell carcinoma | 15 | 12 |  |
| adenocarcinoma | 37 | 28 |  |
| large cell carcinoma | 2 | 4 |  |
| poorly differentiated carcinoma | 7 | 4 | 0.6741 |
| Tumor size |  |  |  |
| <50mm | 36 | 33 |  |
| 50mm≥ | 25 | 15 | 0.3230 |
| Pathology stage |  |  |  |
| I-II | 49 | 36 |  |
| III-IV | 12 | 13 | 0.4934 |

**Table S2. Human 15-LOX1 gene methylation level in NCI-H23 and Bet1A cells treated by PM2.5 and NNK.**
